# Dissociation between behavior and motor cortical excitability before and during ballistic wrist flexion and extension in young and old adults

**DOI:** 10.1371/journal.pone.0186585

**Published:** 2017-10-26

**Authors:** Tibor Hortobágyi, Adinda Mieras, John Rothwell, Miguel Fernandez del Olmo

**Affiliations:** 1 University of Groningen, University of Groningen Medical Center, Center for Human Movement Sciences, Groningen, The Netherlands; 2 Institute of Neurology, University College, London, United Kingdom; 3 Learning and Human Movement Control Group, University of A Coruña, A Coruña, Spain; Nanjing Normal University, CHINA

## Abstract

**Purpose:**

Aging is associated with slow reactive movement generation and poor termination.

**Objective:**

We examined the hypothesis that the build-up of excitability in the primary motor cortex in the agonist muscle to generate ballistic wrist flexion and extension and in the antagonist to stop the movement, is lower and slower in old compared with young adults.

**Methods:**

We measured the size of the motor potentials evoked (MEP) produced by transcranial magnetic stimulation (TMS), background integrated EMG (iEMG), and the MEP:iEMG ratio in healthy young (23 y, n = 14) and old adults' (73 y, n = 14) wrist flexors and extensors as they rapidly flexed or extended the wrist in response to an auditory cue. TMS was delivered at 80% of resting motor threshold randomly in 20 ms increments between 130 and 430 ms after the tone.

**Results:**

Even though old compared to young adults executed the two wrist movements with ~23% longer movement duration and ~15% longer reaction time (both p < 0.05), the rise in MEP:iEMG ratio before the main similar in the two age groups.

**Conclusion:**

These data suggest that an adjustment of current models might be needed to better understand how and if age affects the build-up excitability accompanying movement generation and termination.

## Introduction

Age-related structural and functional changes in the neuromuscular system affect even healthy old adults’ ability to generate and stop voluntary movements [1–6]. However, little is known about the age-related changes in the preparatory mechanisms of motor cortical control of voluntary movement. In self-initiated and reaction time tasks motor cortical activity precedes the agonist electromyographic (EMG) burst by 100 to 400 ms in primates [7, 8]. Reaction time studies using transcranial magnetic stimulation (TMS) showed that the size of motor evoked potential (MEP) increased 20 to 100 ms prior to the agonist EMG burst in healthy young subjects compared with rest, suggesting, akin to the primate data, a rise in motor cortical excitability preceding movement generation [9–15]. However, this is not a universal observation because in simple and choice reaction paradigms there also was a reduction in corticospinal excitability in healthy young adults [16–18] (see also for a review [19]).

Whether and if old age affects the magnitude and time course of the brain activation preceding the generation of a ballistic movement in reaction time tasks is also unclear. The results are inconsistent, as sensorimotor integration measured by electroencephalography (EEG) did not correlate with response speed [20] and MEP size increased in the right hand but decreased in the left hand in the preparatory phase of rapid thumb movements in old compared with young adults [21]. Considering that old compared with young adults’ reactions to visual and auditory cues are substantially slower [20, 22], it is conceivable that the build-up of excitability in the primary motor cortex before the movement is faulty, causing in part the slower reaction.

Age also affects the stopping of voluntary movements. TMS studies suggest an age- related malfunction of GABAergic inhibitory circuits, as motor cortical inhibition is impaired to stop a movement [22–24] and connectivity in white matter tracts predicts this inhibitory dysfunction [25]. Age also seems to affect cortical reciprocal inhibition at rest [26]. These data suggest that the preparatory excitability of the antagonist muscle before it stops the movement is also sub-optimal in magnitude and timing.

We thus examined the hypothesis that the build-up of excitability in the primary motor cortex in the agonist muscle to generate ballistic wrist flexion and extension and in the antagonist to stop the movement, is lower and slower in old compared with young adults. The purpose of the study was to examine the effects of age on the magnitude and timing of motor cortical control of agonist and antagonist wrist muscles in a ballistic movement using a reaction-time paradigm.

## Methods

### Subjects

We recruited healthy young (n = 14, 7M) and old volunteers (n = 14, 7M) by advertisements around the campus and the city area and by word of mouth. Subjects were included in the study if they were healthy based on answers to a health questionnaire, right handed [27], and had a score of ≥ 28 on Mini-Mental State Examination [28]. We quantified the level of physical activity using the Short Questionnaire to Assess Health-Enhancing Physical Activity or SQUASH [29]. Subjects were excluded from the study if they had a history of or at the time of the study had a neurological condition (stroke, Parkinson’s disease, multiple sclerosis, migraine, epilepsy), osteoarthritis, and previous fractures or orthopedic conditions to the upper extremities, and had had contraindications against TMS. Subjects gave written informed consent. The experimental procedures conformed to the declaration of Helsinki and were approved by the Medical Ethical Committee of the University Medical Center Groningen.

Experimental protocol. We collected data in one, 2-hour-long experiment. The skin was prepared for electromyography (EMG) by shaving, scrubbing with fine sandpaper, and cleaning the skin with alcohol over the belly of the right flexor carpi radialis (FCR) and extensor carpi radialis longus (ECR). Subjects sat in a chair with the right elbow flexed to 90° and forearm placed and immobilized in the sponge-padded cuff of a manipulandum in a semi-supinated position with the fingers extended. The center of the wrist joint was aligned with the center of rotation of a shaft connected to a potentiometer mounted on an adjustable stand next to the chair.

The experiment started with peripheral nerve stimulation to determine the maximal compound action potential (Mmax). Mmax was defined as the maximal peak-to-peak amplitude of the M-wave as a response to electrical stimulation of the right radial and median nerve above the elbow. An electrical stimulator delivered the 1.0-ms-long square-wave stimulus (DS7A, Digitimer Ltd, Welwyn Garden City, UK). The stimulation intensity was increased until the peak-to-peak amplitude of the M-wave did not increase any further and then stimulation intensity was increased by an additional 20%. Subjects then performed three, 3-s-long maximal voluntary wrist extensions and flexions (MVCs) with the manipulandum blocked. The EMG data from these trials were used to normalize the magnitude of background EMG activity.

We used an auditory reaction time paradigm to examine motor cortical excitability accompanying the triphasic EMG pattern [15]. Subjects responded to an auditory tone by flexing or extending their wrist “as quick as possible” to a target of 20°. Before the start of data collection subjects practiced the wrist movements 20 times with and without the auditory cue. The start (0°, wrist straight) and target (20° of flexion or extension) positions were displayed on a 1x1 m projection screen 1.5 m away from the subject. After each trial, the subject slowly returned the hand to the start position.

Based on pilot experiments, we tested 17 TMS conditions during the experiment. In one of the 17 conditions, only the tone was presented and in the 16 other conditions a single TMS pulse was delivered in 20 ms increments between 130 and 430 ms after the tone. One testing block contained three times the 17 conditions. In total, 3 blocks of 68 trials for the flexion were administered, followed by 3 blocks of 68 trials for the extension movement, so 204 trials per movement, a total of 408 trials for flexion and extension. The blocks and the order of flexion and extension experiments were randomized. Subjects heard the tone every 5 s (±10%). Subjects rested for 5 minutes between blocks and for 15 minutes between the flexion and extension experiments when they were able to walk around.

### EMG recording

We recorded the EMG activity using 37x27x15 mm, <15 g, wireless, pre-amplified (909x), parallel-bar sensors, affixed to the skin with a four-slot adhesive skin interface (Trigno, Delsys Inc., Natick, MA, USA). The electrodes recorded with a bandwidth of 20–450 Hz, channel noise <0.75 μV, and common mode rejection ratio >80 dB. The EMG signals and potentiometer were sampled at 4kHz (Power 1401, Cambridge Electronics Design, Cambridge, UK), acquired online and stored by software installed on a personal computer for offline analysis (CED Power 1401 and Signal version 5.0, Cambridge Electronics Design, Cambridge, UK).

### TMS

We generated motor evoked potentials (MEPs) in the FCR or ECR with single pulse TMS delivered by a Magstim 200 stimulator (Magstim Company Ltd, Dyfed, UK). To elicit MEPs in the right FCR or ECR, we used a figure of eight-shaped coil (loop diameter, 90 mm) held over the optimal stimulation spot of the left primary motor cortex (M1) with the handle pointing backwards at -45° away from the sagittal plane. The site where stimuli of slightly supra-threshold intensity consistently produced the largest MEPs with the steepest negative slope in the right FCR or ECR muscle (referred to as “motor hotspot”) was marked with a black wax pencil by drawing a line following the anterior bifurcation of the coil and a straight line as an extension of the handle at the top of the coil on a white cap placed on the head of the participant, indicating the orientation of the coil handle. The current in the coil flowed counter clockwise (viewed from above). We set the stimulation intensity at 80% of resting motor threshold (rMT) because such intensity was successfully used to probe corticospinal excitability in reaching, grasping, precision lift, and also in a reaction time task [15, 30]. Also, the baseline is more variable with supra-threshold than sub-threshold TMS, therefore small changes in corticospinal excitability are easier to detect with sub-threshold TMS [9].

### Control experiment

Using the TMS protocol and setup, we also evoked H reflexes by electrically stimulating the median nerve just above the elbow crease in healthy young subjects (n = 5) who did not participate in the TMS experiments. Pulses of 1-ms duration were delivered through a pair of moist gauze covered button electrodes placed 2.5 cm apart with the cathode located proximal to the anode (Digitimer DS7, Welwyn Garden City, Hertfordshire, UK). We completed a recruitment curve by increasing the stimulation intensity in 2 mA steps to evoke an H reflex and Mmax. We then used a stimulation intensity that evoked 50% of Hmax in the 17 states described for the TMS experiment during wrist flexion when the FCR acted as the agonist and during wrist extension when the FCR acted as the antagonist. The order of flexion and extension was randomized between subjects.

### Data analysis

To characterize behavioral differences between young and old adults, we determined range of motion, movement duration, peak velocity, reaction time, electromechanical delay, onset of agonist EMG activity after the tone, onset of antagonist EMG relative to onset of the agonist, antagonist coactivity as a percent of MVC and antagonist coactivity as a ratio of agonist EMG activity to antagonist EMG activity. Profiles of the EMG activity were baseline corrected and full wave rectified. Joint position data were differentiated to obtain wrist angular velocity. Electromechanical delay was computed as the difference between onset of agonist and movement onset with the latter being the time point when the velocity exceeded two standard deviations (SD) from baseline, computed by a script in Signal, and confirmed by visual inspection. Onset of agonist and antagonist activity were determined when the activity exceeded two SDs from baseline. We also used the 2SD-threshold to determine when velocity differed from zero.

We quantified the magnitude of the MEPs by calculating the peak-to-peak voltage of the evoked responses in each trial. We also determined the amount of the background EMG activity by computing the area under the EMG envelope, i.e., iEMG, between 5 to 13 ms, after the TMS artifact and the onset of the MEP. The MEP:iEMG ratio quantified the influence of the background EMG activity on MEPs [15]. In each subject individual MEPs were referenced to movement onset. The referenced MEPs were then sorted into 10-ms bins and averaged. In each subject and in each bin, the MEPs and the iEMG activity were normalized to the maximal compound action potential (Mmax) or to maximal iEMG measured during voluntary wrist extension or flexion. Even though each subject performed a total of 204 trials, in 4.2% of the cases there were no MEPs in a given 10-ms bin. Therefore, in the main analysis we averaged adjacent 10-ms bins into 9, 20 ms bins.

### Statistical analyses

We report the data as mean ±SD (SPSS, v. 20.0). We compared the behavioral data between the two age groups with an unpaired t-test. The main analysis involved an age group (young, old) by time (9, 20-ms bins) analysis of variance with repeated measures on time. The ANOVA was used without an age grouping factor for the H reflex in the control experiment. To determine if the timing of MEP and iEMG activity in the agonist and antagonist muscle differed within each group, we performed an ANOVA with repeated measures on time and type of EMG measure (MEP, iEMG). For significant interactions, we used Tukey’s post hoc contrasts to determine the means that were significantly different at p < 0.05.

## Results

**Fig 1:**
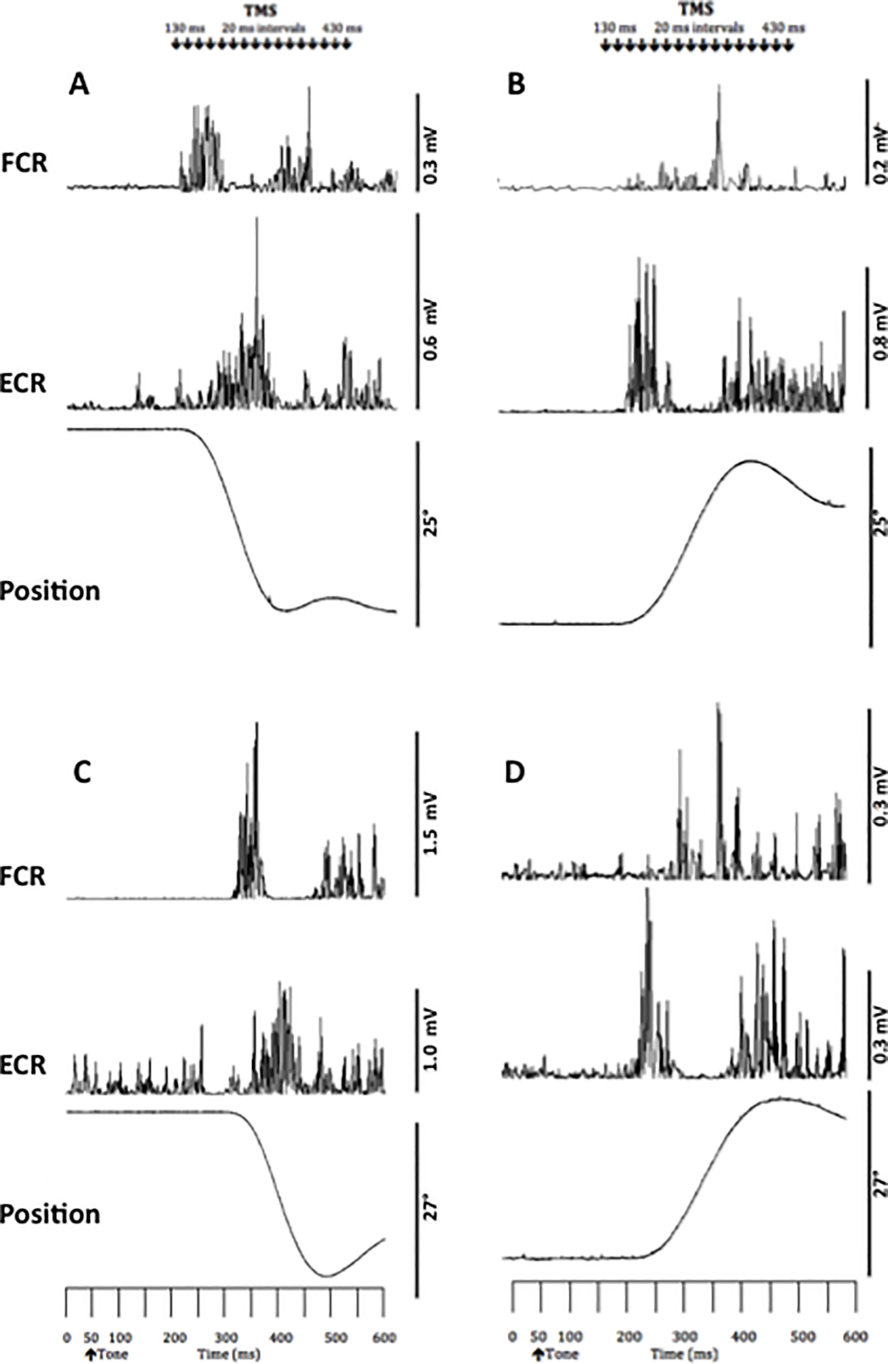
Typical recording. Representative example of rectified EMG activity of the flexor carpi radialis (FCR) and extensor carpi radialis longus (ECR) muscles during wrist flexion (A, C) and extension movements (B, D) in one young (A, B) and one old (C, D) subject. The lowest tracing within each panel is the wrist joint position data. Note the triphasic pattern of EMG activity associated with movements in both directions. The arrows at the top of the figure show the times when TMS was applied across. The auditory tone was presented at 50 ms.

Table 1 shows the age-normal characteristics of the subjects. Fig 1 shows representative examples of wrist flexion and extension in a young and old adult. Table 2 shows that old compared to young adults executed the two wrist movements with ~23% longer movement duration and ~15% or ~28 ms slower reaction time (p < 0.05).

**Table 1:** Characteristics of the subjects.

Variable	Young	Old
n (M/F)	14, 7/7	14, 7/7
Age, y	22.5 (1.70)	72.9 (3.52)
Mass, kg	70.7 (14.62)	76.2 (12.21)
Height, m	1.76 (0.09)	1.73 (0.08)
BMI, k·/m^-2^	22.71 (3.11)	25.54 (4.21)
MMSE	-	28.9 (1.07)
SQUASH	11.0 (3.13)	13.5 (6.27)

Values are mean (±SD)

BMI, body mass index

MMSE, Mini Mental State Examination (>27 cognitively healthy)

SQUASH, Short Questionnaire to Assess Health-Enhancing Physical Activity

**Table 2:** Behavioral data of rapid wrist flexion and extension in response to an auditory cue in young and old adults.

Variable	Young	Old	Δ,Abs.	Δ,%	P-value
ROM, °					
Flexion	24.9 (3.6)	27.2 (3.9)	2.3	9.2	0.118
Extension	25.2 (3.3)	27.3 (2.9)	2.2	8.6	0.073
Movement dur., s					
Flexion	0.221 (0.04)	0.282 (0.06)	0.06	27.5	0.010*
Extension	0.241 (0.04)	0.283 (0.04)	0.04	17.7	0.013*
Peak velocity, °/s					
Flexion	112.4 (39.5)	104.8 (16.1)	-7.6	-6.8	0.513
Extension	178.1 (31.7)	183.7 (33.1)	5.6	3.1	0.652
Reaction time, ms					
Flexion	187.9 (21.9)	222.6 (29.6)	34.6	18.5	0.002*
Extension	189.1 (29.0)	210.8 (30.8)	21.7	11.4	0.066
EMD, ms					
Flexion	14.8 (8.1)	20.4 (8.9)	5.6	37.9	0.095
Extension	21.3 (11.6)	21.1 (10.8)	-0.15	-0.7	0.065
Onset of agonist, ms					
Flexion	173.1 (17.5)	202.2 (33.2)	29.1	16.8	0.009*
Extension	167.8 (28.2)	189.7 (31.5)	21.8	13	0.268
Onset of antag., ms					
Flexion	62.9 (25.3)	72.3 (39.7)	9.4	15	0.462
Extension	101.2 (25.2)	116.3 (43.1)	15.2	15	0.973
Antag. coact., %MVC					
Flexion	27.0 (16.4)	32.5 (14.7)	5.6	20.6	0.354
Extension	7.4 (4.4)	7.6 (5.1)	0.22	2.91	0.907
Antag. coact., ANT/AG %					
Flexion	86.3 (61.3)	93.3 (53.3)	7	8.1	0.749
Extension	21.4 (14.5)	16.7 (7.9)	-4.8	-22.2	0.292

Values are mean (±SD).

Δ, Abs., absolute difference between young and old computes as (Y-O)*(-1)

Δ, %, percent difference between young and old, computed as ((Y-O)/Y)*(-100)

ROM, range of motion

Onset of agonist, start of the agonist main EMG burst after the auditory cue

Antagonist coactivity, % MVC, surface EMG activity of the antagonist during the main agonist EMG burst expressed as a percent of EMG measured during a maximal voluntary isometric contraction in the neutral position

Antagonist coactivity, ANT/AG% is the ongoing antagonist surface EMG activity during the agonist EMG burst, expressed as a percentage of the EMG activity

* p < 0.05 between Young and Old groups

**Fig 2:**
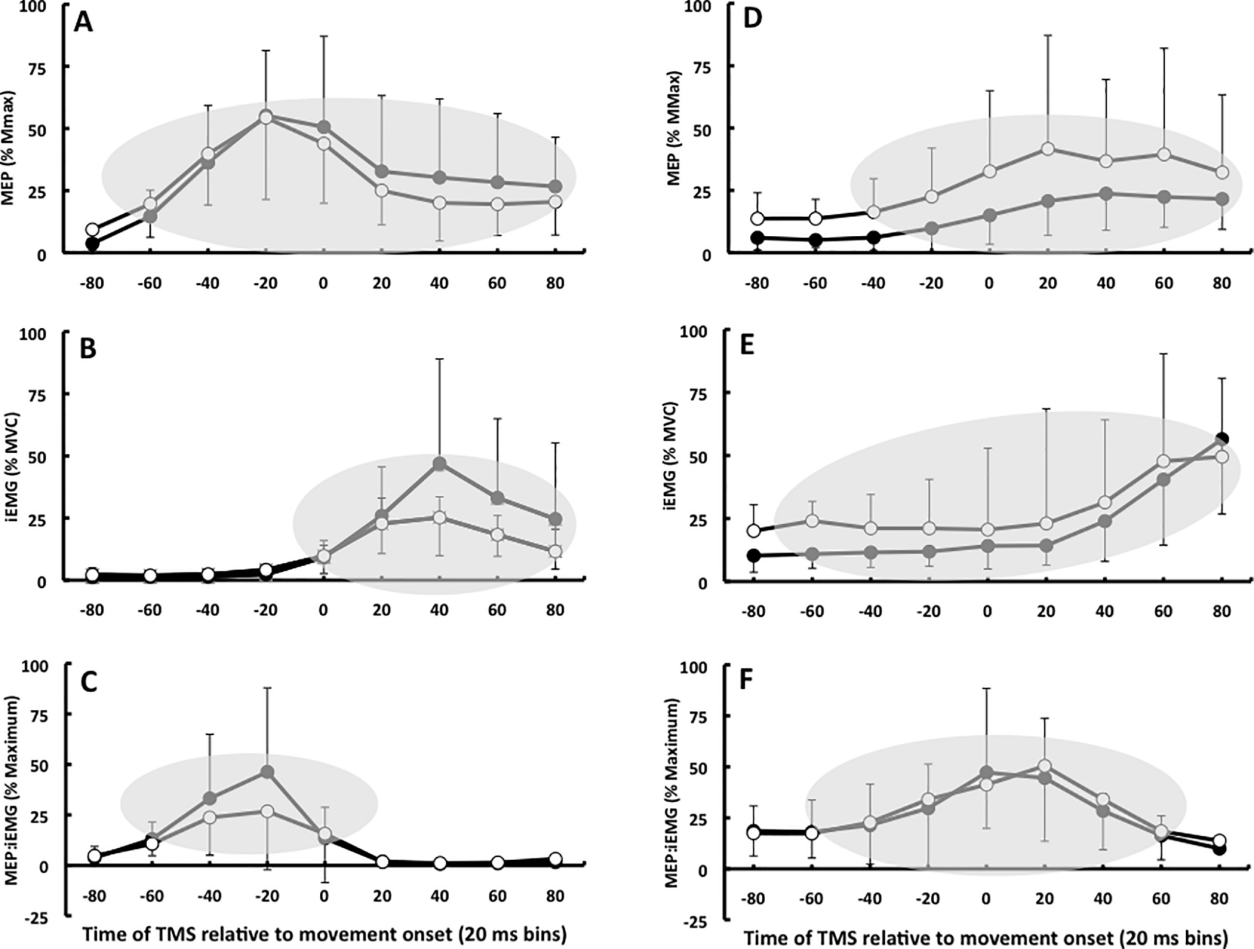
Time course data. Time-varying changes in the amplitude of the MEPs (A,D), IEMG (B,E), and MEP:IEMG (C,F) ratios in the flexor carpi radialis (A,B,C) and extensor carpi radialis longus (D,E,F) during wrist flexion in response to an auditory cue in healthy young (n = 14, filled symbols) and old (n = 14, unfilled symbols) adults. Symbols within shaded areas are different from zero (p < 0.05) but not different between young and old adults (p > 0.05). Values are normalized to Mmax (A,D), MVC (B,E), and to maximum response within subjects (C,F). Increases in agonist MEPs preceded the agonist burst, causing the increase in MEP:IEMG ratio. Holding the wrist in an extended position in preparation for wrist flexion produced a steady background IEMG and a moderate increase in the ratio prior to movement (time 0), suggesting a mild elevation in excitability. Error bars denote ±SD.

**Fig 3:**
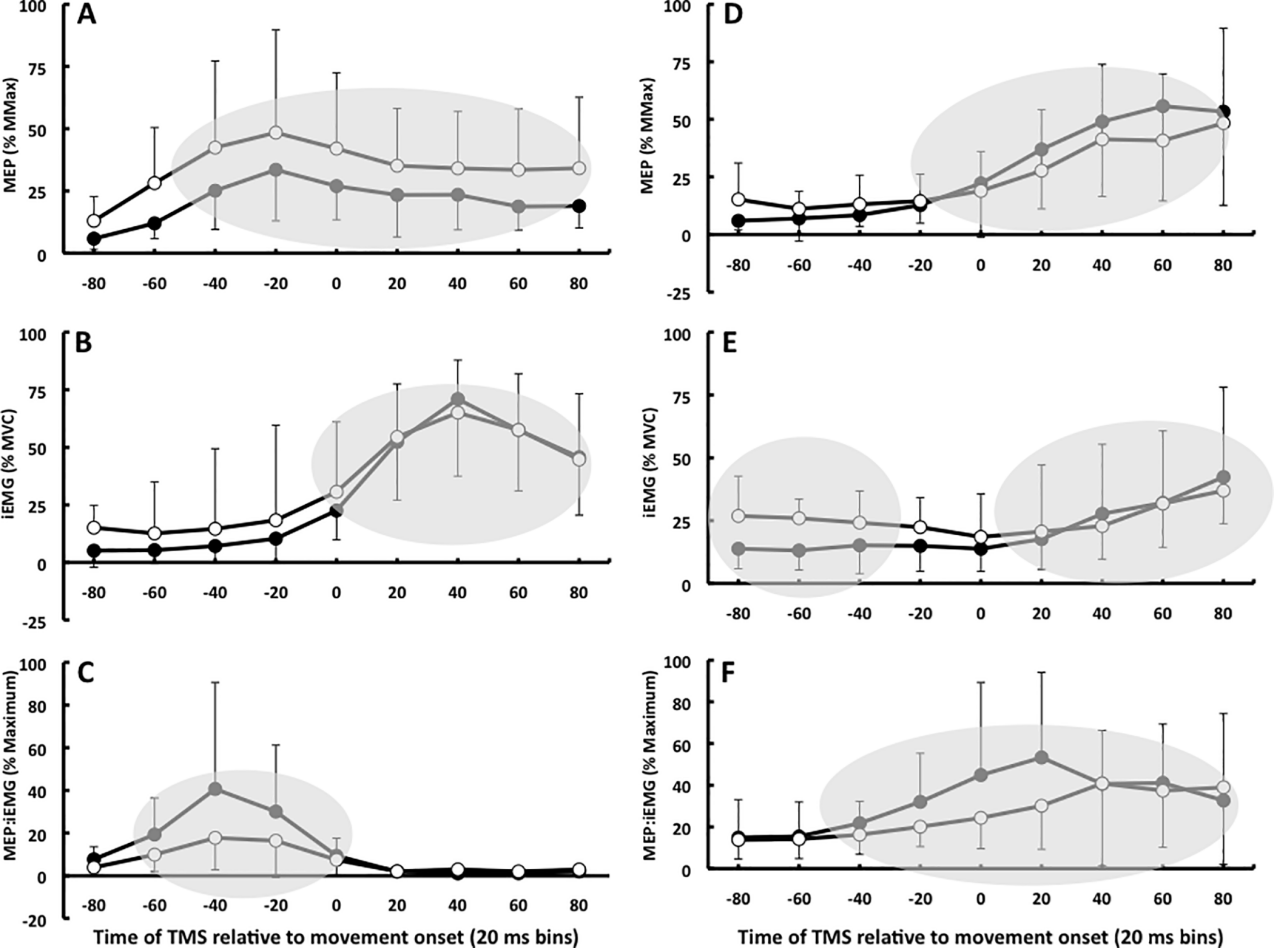
Time course data. Time-varying changes in the amplitude of the MEPs (A,D), IEMG (B,E), and MEP:IEMG (C,F) ratios in the extensor carpi radialis longus (A,B,C) and flexor carpi radialis (D,E,F) during wrist extension in response to an auditory cue in healthy young (n = 14, filled symbols) and old (n = 14, unfilled symbols) adults. Symbols within shaded areas are different from zero (p < 0.05) but not different between young and old adults (p > 0.05). Values are normalized to Mmax (A,D), MVC (B,E), and to maximum response within subjects (C,F). Increases in agonist MEPs preceded the agonist burst, causing the increase in MEP:IEMG ratio. There were moderate increases in the ratio prior to movement (time 0), suggesting a mild elevation in excitability. Error bars denote ±SD.

Fig 2 shows the time-varying changes in the amplitude of the MEPs, IEMG, and MEP:IEMG ratios in the agonist flexor carpi radialis and antagonist extensor carpi radialis longus during wrist flexion in response to an auditory cue in the two age groups. While MEPs, iEMGs, and their ratios differed from zero during wrist flexion (Fig 2A–2F, shaded areas), the age main effects and the age by time bin interactions were not significant in the agonist FCR and antagonist ECR (range of values for F1,26 = 0.1 to 3.5 and for p = 0.840 to 0.074). Likewise in Fig 3A–3F, none of the age main effects and age by time bin interactions were significant during wrist extension in the agonist ECR and antagonist FCR (range of values for F1,26 = 0.1 to 3.7 and for p = 0.736 to 0.065). Figs 2C and 3C show that corticospinal excitability started to increase 40–60 ms prior to start of wrist flexion and extension movement with the iEMG peaking in both muscles around 40 ms after movement onset.

**Fig 4:**
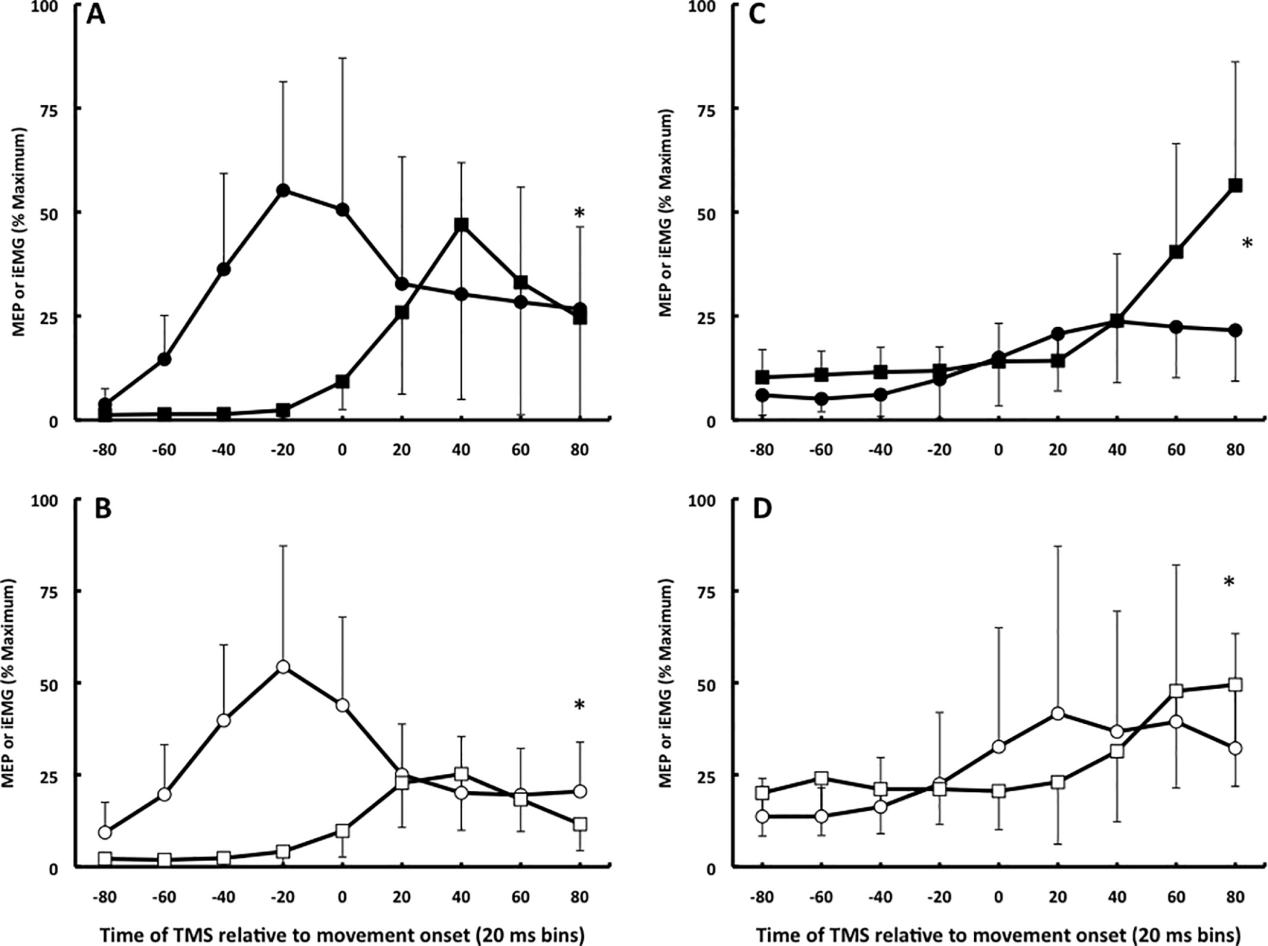
Time course data. Time-varying changes in the amplitude of the H reflex (filled symbol) and iEMG (open symbol) normalized to the maximal compound action potential (Mmax) in the flexor carpi radialis acting as the agonist (A) and antagonist (B) during ballistic wrist flexion. Error bars denote ±SD, n = 5.

In the control experiment (n = 5, age 22.8, 2M) the size of the peak-to-peak amplitude of the maximal H reflex and M wave evoked by stimulating the median nerve at the elbow at rest, respectively, was 1.6 mV (±0.3) and 4.2 mV (±1.8). Fig 4 shows that the H reflex normalized to Mmax in the FCR showed time-varying changes when acted as an agonist during ballistic wrist flexion (F8,32 = 12.3, p = 0.007, panel A) but did not show a time-varying pattern across the 9 time bins when the FCR acted as antagonist during ballistic wrist extensions (F8,32 = 1.1, p = 0.663, panel B). While MEPs preceded the agonist iEMG burst (Figs 2 and 3), the H reflex and iEMG changed in parallel (Fig 4).

## Discussion

We examined the effects of age on corticospinal excitability at different times before and during the triphasic EMG pattern accompanying rapid wrist flexion and extension. Even though old compared to young adults executed the two wrist movements with ~23% longer movement duration and ~15% longer reaction time (both p < 0.05), the rise in MEP:iEMG ratio before the main agonist and antagonist EMG burst was similar in the two age groups.

We observed a distinct rise in corticospinal excitability before the main agonist burst during wrist flexion and extension (Figs 2C and 3C). The rise occurred in a range of 20–60 ms prior to the main agonist burst [15], closer to the burst than the 80–100 ms reported in other studies [9–13]. The shorter lead-time is probably related to referencing MEPs to agonist EMG onset within each trial instead of the average onset of the main agonist burst computed across many trials, a less accurate method. The sharp rise in the MEP:iEMG ratio before the main agonist burst lasted for ~40–60 ms in both muscles and age groups (Figs 2C and 3C) but the rise in the H wave:iEMG ratio did not precede but paralleled the main agonist burst (Fig 4). The iEMG reflects activity of motoneurons discharging action potentials, whereas the descending input by TMS excites yet unrecruited subliminal fringe motorneurons. It is likely that TMS excited this unrecruited portion of the motoneuron pool, causing the rise in the MEP:iEMG ratio emanating most likely from cortical sources. A cortical vs. a spinal origin of the rise in excitability is also more likely because we used sub-threshold, low-intensity magnetic pulses which produce MEPs through monosynaptic corticospinal connections without presynaptic control of the corticomotoneuronal synapse [31].

Even though old compared to young adults executed the two wrist movements with ~23% longer movement duration and ~15% longer reaction time (both p < 0.05), the rise in MEP:iEMG ratio before the main agonist burst was similar in the two age groups. We referenced our data to movement onset, normalized MEPs to iEMG, and used sub-threshold TMS intensity while previous studies referenced the data to the “GO” signal, normalized premotor MEPs to MEPs measured at rest or at the warning signal, and used supramaximal TMS intensity (110% of resting motor threshold), making comparisons of the present data with those published previously difficult [16,17]. Depending on how the data were normalized, in contrast to our data, there were no changes in excitability in the premotor phase in young and an increase in excitability in old adults’ right thumb but a depression in the left thumb [17] or both age groups showed, in contrast to our data, a suppression of premotor excitability in the index finger [16]. These authors suggested that old adults’ motor slowing was related to the degree of suppression in premotor corticospinal excitability.

We designed our experiment based on the concept that old adults’ slowed reaction to the auditory cue would necessitate a slow and early ramp up of premotor cortical excitability. But we did not find this. Our data point to a model according to which once the buildup of motor cortical excitability before the agonist EMG burst reaches a threshold, this threshold acts like a switch and causes the immediately release of corticospinal activity, resulting in a similar excitability patterns in the two age groups. Alternatively, motor cortex activity ramps up, but then needs an external signal to release the descending activity [32]. Whichever is true, the data favor a model where the time taken for preparation to move involves a different mechanism than the trigger to move. Preparation is slow in the elderly, but the trigger is unimpaired, and the commands, when ready, are released as normal. Indeed, Parkinson’s patients execute a simple reaction time task with the prolonged period of premotor facilitation compared with age-matched healthy controls [33].

Previous studies have not yet examined the effects of age on motor cortical excitability preceding the antagonist EMG burst accompanying the stopping of a ballistic movement. Compared with the MEP:iEMG ratios accompanying the agonist burst, the rise in this ratio was less steep and 20–40 ms more prolonged in both muscles and age groups (Figs 2F and 3F), consistent with previous data in young adults [15]. Because old adults tend to execute voluntary movements with a heightened antagonist muscle coactivation, exhibit, at least at rest, aberrant cortical reciprocal inhibition [26], and have deficits in motor cortical inhibition needed to stop a motor act [22–24], we speculated that motor cortical excitability of the antagonist muscle would rise more steeply before the antagonist EMG burst and this excitability would decay more slowly in old compared with young adults. Again, we did not find this nor did we find an increase in antagonist EMG activity (Table 2). Our old participants were highly fit and healthy and health status can be a powerful experimental element because absent or inconsistent age effects have been observed in a number of experimental conditions and measures, including TMS outcomes [34,35], movement variability [36,37], and motor learning [38–40].

In conclusion, in a highly stereotypical ballistic wrist flexion and extension task, we found no evidence for age affecting the time-varying motor cortical control of wrist muscles. The data suggest that an adjustment of current models might be needed to better understand how and if age affects the build-up excitability accompanying movement generation and termination. Certainly our old participants’ good health and the highly programmed pattern of muscle activity could have minimized any age effects. These data add to the body of evidence as to how under certain conditions neural control of voluntary movement remains well preserved in old age.

## Supporting information

S1 File Contains the individual subject data. (XLSX)
